# Neuroimaging and neuromodulation of invisible symptoms in multiple sclerosis

**DOI:** 10.3389/fnhum.2024.1376095

**Published:** 2024-02-22

**Authors:** Samar S. Ayache, Moussa A. Chalah

**Affiliations:** ^1^Department of Neurology, Gilbert and Rose-Marie Chagoury School of Medicine, Byblos, Lebanon; ^2^Institut de la Colonne Vertébrale et des NeuroSciences (ICVNS), Centre Médico-Chirurgical Bizet, Paris, France; ^3^EA4391 Excitabilité Nerveuse and Thérapeutique, Université Paris Est Créteil, Creteil, France; ^4^Department of Clinical Neurophysiology, DMU FIxIT, Henri Mondor University Hospital, Assistance Publique-Hôpitaux de Paris (APHP), Creteil, France; ^5^Pôle Hospitalo-Universitaire Psychiatrie Paris 15, GHU Paris Psychiatrie et Neurosciences, Hôpital Sainte-Anne, Paris, France

**Keywords:** multiple sclerosis, fatigue, depression, anxiety, brain stimulation, rTMS, tDCS, ECT

## 1 Introduction

Multiple Sclerosis (MS) is an autoimmune disease that affects the central nervous system via several mechanisms (i.e., demyelination, synaptopathy, and neurodegeneration) (Jakimovski et al., 2023). It is thought to involve an interaction between (epi)genetic and environmental factors, it concerns 2.8 million individuals worldwide, and frequently occurs in young adults with female predominance, constituting the most common non-traumatic cause of disability in this age group. MS could result in several clinical manifestations with multiple consequences (psychological, physical, social and professional) and an altered quality of life for patients and their informal caregivers, as well as economic and societal burdens (Jakimovski et al., 2023).

MS is considered a “multiple disconnection syndrome” due to lesions disrupting the connectivity within several networks leading to the appearance of various symptoms. Patients with MS (PwMS) can suffer from motor, sensory, and cerebellar symptoms, among others (Jakimovski et al., 2023). They can also exhibit other manifestations that are prevalent and debilitating, yet they are still overlooked and sometimes difficult to assess and manage. These manifestations, also referred to as hidden, silent, or invisible symptoms (to others), include fatigue, affective manifestations, cognitive deficits, pain, and sleep disorders (Lechner-Scott et al., 2019).

To start, up to 90% of PwMS suffer from fatigue, a worrisome and complex manifestation with multifactorial etiology (primary fatigue linked to underlying neural mechanisms vs. secondary related to a plethora of comorbidities, medical and/or iatrogenic factors) and with multiple facets (physical, cognitive, psychosocial) (Ayache and Chalah, 2017). MS fatigue could imply either a general perception (trait) that could get exacerbated in some clinical and environmental conditions or a reversible decline in motor or cognitive performance (state or ‘fatigability').

In addition, affective disorders have a lifetime prevalence of 95% in PwMS, with anxiety and depression being the most common manifestations (Chalah and Ayache, 2017a; Filser et al., 2023; 41% vs. 5–59%, respectively).

Also, cognitive impairment seems to occur in 34%-65% of patients including “cold” cognitive domains (i.e., attention, learning, memory, executive functions) but also “hot” domains that are gaining more interest. In this context, we can cite social cognition (Benedict et al., 2020) and alexithymia. The latter refers to the inability of an individual to identify and describe their own emotions, and their tendency to adopt externally oriented thinking. This difficulty could affect 10–53% of PwMS (Chalah and Ayache, 2017b).

Moreover, 29–86% of PwMS seem to suffer from chronic pain, most commonly the central neuropathic type involving the lower limbs (Ayache and Chalah, 2018). Furthermore, 53.6–74% of PwMS can be affected by sleep disorders that could hamper their quality of life and might contribute to relapses via oxidative stress if left untreated (Foschi et al., 2019).

The abovementioned manifestations can occur anytime in PwMS, some can herald a relapse onset or even sign the beginning of the disease. Interestingly, these symptoms were suggested to co-occur in PwMS (Ayache and Chalah, 2020; Chitnis et al., 2022), supporting the notion of a “symptoms cluster” in this clinical population.

The components of such a cluster share common underlying mechanisms (neuroanatomical and functional cerebral substrates, neurochemical correlates, proinflammatory and neuroendocrine underpinnings), they usually have bidirectional interactions, and they might engender cumulative or synergistic effects on patients' quality of life, adherence to therapeutics, and clinical outcomes (Ayache and Chalah, 2020; Chitnis et al., 2022). Therefore, a thorough understanding of these symptoms would help further characterize their common and distinct neurobiological pathways and subsequently suggest putative therapeutic targets.

Nowadays, these symptoms are mainly assessed using subjective scales that suffer from some pitfalls (e.g., social desirability, recall bias), which emphasizes the importance of identifying objective biomarkers that could take into account the multidimensional nature of the concerned manifestations, and might have good predictive values. Besides assessment-related difficulties, the available pharmacological options are challenged by their side effects and the extent of their clinical benefits that are—at their best—modest.

In this context, functional and structural neuroimaging and related technologies [e.g., magnetic resonance imaging (MRI), positron emission tomography (PET), and transcranial magnetic stimulation (TMS)] might constitute convenient tools to solve the current difficulties and unravel the neural bases of the symptoms in question. Here, the current findings on the application of these techniques in MS hidden symptoms are discussed along with future perspectives that will help move forward in exploration and neuromodulation.

## 2 Neuroimaging and neuromodulation of silent symptoms

### 2.1 Neuroimaging and silent symptoms

Although some of these symptoms could be attributed sometimes to the chronic, unpredictable, and incapacitating nature of the disease (i.e., response to stress, loss of function due to disease relapse or progression, fear of the latter), they might stem from specific cerebral abnormalities as suggested by a growing body of literature. Symptoms seem to have sharable neural substrates (e.g., abnormalities affecting the frontal lobe regions or tracts), but also distinct pathophysiological pathways.

Regarding fatigue, despite the disparities that could be noticed across the available radiological studies, a thorough analysis suggests that many of the observed anatomical and functional abnormalities involve a “cortico-striato-thalamo-cortical loop”, mostly incriminating frontoparietal regions, thalami and basal ganglia, among others (Ayache and Chalah, 2017).

Also, based on structural MRI studies, depression and anxiety symptoms seem to be correlated with frontal abnormalities in some works (Lin et al., 2013; Gobbi et al., 2014; Pravatà et al., 2017). The correlates of depression extend to include temporal, parietal, and limbic abnormalities (Bakshi et al., 2000; Zorzon et al., 2001, 2002; Feinstein et al., 2004; Van Geest et al., 2019). In addition, coupling PET with functional MRI has permitted the observation of a relationship between MS depressive symptoms and decreased functional connectivity within the monoaminergic networks, in line with the general monoamine hypothesis of depression (Carotenuto et al., 2023; Mistri et al., 2023). Similar to depression, anxiety seems to incriminate some structural abnormalities, such as septo-fornical damage (Palotai et al., 2018), limbic lesions (Hillyer et al., 2023), dorsal prefrontal thinning, and an altered amygdala-hippocampal-prefrontal functional network (Ellwardt et al., 2022); a network previously identified in anxiety circuitry.

As for alexithymia, few works are available suggesting a correlation between this construct and corpus callosum, thalamic, and brainstem atrophy (Chalah et al., 2020a; Capet et al., 2021).

Moreover, cognitive impairment seems to be associated with an involvement of neocortical, hippocampal and deep gray matter, including the thalamus and/or altered connectivity between the involved gray matter hubs (Benedict et al., 2020).

Pain *per se* is thought to arise from spinothalamic nociceptive pathways lesions and neuroimaging data suggest an involvement of brainstem lesions in MS pain (Seixas et al., 2014).

As for sleep, although some disorders (i.e., insomnia) seem to be related to physical (e.g., pain, sphincteric disorders) or psychological symptoms (e.g., anxiety, depression), other problems could be associated with MRI abnormalities: brainstem lesions in sleep-related breathing disorders, hypothalamic lesions in narcolepsy, and infratentorial lesions in restless leg syndrome or period limb movement disorders (Foschi et al., 2019). In recent functional MRI studies, correlations were found between poor sleep quality or insomnia and decreased functional connectivity of the left intraparietal sulcus and the thalamus, respectively (Van Geest et al., 2017; Ruiz-Rizzo et al., 2022).

It is worth noting that besides neuroimaging, noninvasive brain stimulation techniques—particularly TMS—have been used to explore the relationship between corticospinal excitability parameters and some of these symptoms (Stampanoni Bassi et al., 2020). By adopting a single- or double-pulse paradigm and applying magnetic stimuli over the primary motor cortex, TMS induces motor-evoked potentials and permits the acquisition of several parameters that reflect the function of intracortical and interhemispheric circuits. Based on the very few available TMS studies in MS, fatigue and alexithymia seem to be associated with enhanced intracortical GABAergic inhibitory activity. Anxiety (but not depression) was correlated with an altered interhemispheric inhibition; and cognitive impairment (i.e., verbal memory) was linked to a hampered cortical cholinergic inhibition. No studies are available on pain or sleep (Chalah et al., 2020b; Stampanoni Bassi et al., 2020).

### 2.2 Neuromodulation and silent symptoms

The prevalence of these symptoms and their putative neural signatures make them an appealing target for neuromodulation. Here, electroconvulsive therapy (ECT)—the oldest intervention compared to repetitive TMS (rTMS) and transcranial electrical stimulation (tES)—consists of applying a brief pulsed current to the brain through two electrodes and under general anesthesia to trigger a seizure that underlies the clinical benefits via neurophysiological, neurochemical and neuroplastic mechanisms. Despite its availability, only few case reports have been published on this matter (Steen et al., 2015). ECT appears to be generally safe (neurological deterioration reported in only very few cases) and efficacious when applied to treat psychiatric manifestations in PwMS (Steen et al., 2015). Further investigation is appreciated.

Compared to ECT, rTMS and tES do not induce seizures and do not require anesthesia. Besides the previously stated application of TMS as an exploration tool, rTMS applied over a cerebral area using low (≤ 1 Hz) or high (≥5 Hz) frequencies exerts inhibitory vs. excitatory effects, respectively. However, one should keep in mind that the effects are more complex and would depend on several factors. rTMS could result in short- and long-term changes depending on the applied protocols. From a mechanistic point of view, rTMS could result in neuroplastic changes (e.g., long-term potentiation-like and long-term depression-like plasticity) and its effects might involve neurotrophic, neuroimmune, and neuroendocrine factors. This could be paralleled by changes in oscillatory brain activity, corticospinal excitability parameters, regional brain volumes or connectivity, or metabolic activity when coupling the technique with electroencephalography, motor evoked potentials and neuroimaging (Kricheldorff et al., 2022).

As for tES, particularly transcranial direct current stimulation (tDCS), it consists of applying a low-intensity current over the scalp generally using two electrodes (an anode and a cathode). Other tES techniques consist of applying an alternating current (tACS) or a random noise (tRNS). tDCS effects depend on electrode polarity and is thought to exert depolarization and hyperpolarization of the resting membrane potential under the anode and the cathode, respectively (Woods et al., 2016; Lefaucheur et al., 2017). tDCS could exert acute/short as well as long term effects, which might result from different mechanisms. While the former could involve changes in membrane polarization, neurotransmitters releases, and spike-timing-dependent plasticity, the latter could involve neurogenesis, synaptic plasticity and cortical reorganization (Kricheldorff et al., 2022). Like rTMS, tDCS effects could appear as changes in neurophysiological and neuroimaging measures.

Recent reviews and metanalyses on tDCS application in MS symptoms have shown a major focus on MS fatigue with a significant symptom reduction when targeting the prefrontal or sensorimotor cortices (Ayache and Chalah, 2018). tDCS also appears to decrease pain (targeting left dorsolateral prefrontal or primary motor cortex), alleviate psychiatric symptoms (mainly targeting prefrontal regions) when pooling anxiety and depression data together, and have a trend toward cognitive enhancement (mainly targeting prefrontal regions). Only a single pilot tDCS study has addressed sleep quality in PwMS by applying prefrontal stimulation and showed an improvement in subjective daytime sleepiness, but not objective sleep parameters (for reviews see Uygur-Kucukseymen et al., 2023). Besides tDCS, tRNS has been applied but to a lesser extent and the very limited available data suggest no effects on the studied outcomes (for reviews see Uygur-Kucukseymen et al., 2023). As for tACS, a recent study using a single session suggests potential cognitive benefits (Hsu et al., 2023).

Compared to tES, fewer studies have applied rTMS in PwMS. Nevertheless, the majority were interested in fatigue and showed significant benefits (Ayache et al., 2022), one single study found promising antidepressive effects (Ahmadpanah et al., 2023), and single studies yielded negative outcomes in pain and cognition (for reviews see, Uygur-Kucukseymen et al., 2023).

## 3 Discussion

The available data altogether have increased the scientific insight into silent MS symptoms. Data are promising, albeit scarce, and faced with several limitations. Heterogeneity across the studies could be attributed to several variables: small sample sizes, clinical and sociodemographic differences, as well as methodological discrepancies (i.e., study design, assessment tools (scales vs. standardized clinical evaluations), neuromodulation protocols). In addition, some interesting notions -namely brain and cognitive reserves- are worth considering to further explain the observed divergences (Sumowski et al., 2013). Briefly, one could perceive the brain reserve and the cognitive reserve as the “hardware” and the “software” of the brain, respectively (Stern et al., 2019). While brain reserve is considered a “physical trait” reflected by the brain volume (i.e., associated with the number of neurons and synapses), the cognitive reserve is associated with intellectual enrichment and lifetime experiences (i.e., premorbid intelligence quotient, education, professional experience, leisure activities) (Stern et al., 2019). These reserves might act as a mediator between the extent of tissue damage and the clinical decompensation, particularly upon the exhaustion of compensatory mechanisms. They might explain why PwMS with similar radiological abnormalities exhibit or do not have clinical symptoms and could also partially explain differences in neuroimaging correlates of the concerned symptoms across the studies. For instance, considering patients with similar radiological measures, those with higher brain and/or cognitive reserves would be clinically intact or mildly impaired compared to those with lower reserves.

With regards to the symptoms cluster exploration, future large-scale multidimensional works would be pertinent to develop objective symptom markers, predict symptom occurrence and treatment response, and finely decipher the symptoms' overlapping and separate paths. For instance, in one work, combining neuroimaging and blood biomarkers (neurofilament light chain) helped improve the diagnostic accuracy of cognitive decline in MS (Brummer et al., 2022). In another work, early microstructural changes (thalamic, amygdalar, hippocampal) predicted the later onset of depressive symptoms (Riemer et al., 2023). In a third work, frontostriatal damage predicted the pharmacological resistance in MS fatigue (Palotai et al., 2023).

As for neuromodulation, the available techniques are safe and their effects merit to be replicated and optimized in future randomized controlled trials that include long-term outcomes. Admitting the dose-response relationship observed when using neuromodulation (Hutton et al., 2023), one way to improve the treatment efficacy would be by increasing the number of sessions and protocol duration. Another factor that is worth improving would be the cerebral targeting method. Here, neuronavigation-guided rTMS using individual functional MRI data appears to be a promising approach for treating depression (Fox et al., 2012; Caulfield et al., 2022; Lynch et al., 2022), and is worth adopting in MS research. In addition, combining neuromodulation with other interventions might engender cumulative or synergistic effects. The choice of interventions would depend on the symptom in question and could include cognitive training (Benedict et al., 2020), neurofeedback (Ayache et al., 2021), psychotherapies (Sesel et al., 2018), physical exercise (Muñoz-Paredes et al., 2022), neurobiological methods (Hertenstein et al., 2021), interoceptive technologies (Schoeller et al., 2024), among others. Finally, coupling neuroimaging and neuromodulation would help unveil the underlying neuromodulation mechanisms. Such investigations would pave the way for developing a patient-tailored and multidisciplinary approach.

## Author contributions

SA: Conceptualization, Formal analysis, Methodology, Supervision, Validation, Writing—original draft, Writing— review and editing. MC: Conceptualization, Formal analysis, Methodology, Supervision, Validation, Writing—original draft, Writing—review and editing.
